# At What Age Should a Baby Be Vaccinated?

**Published:** 1913-03-08

**Authors:** 


### MATERNITY AND MOTHERING.

(Inquiries and Correspondence are invited.)

### At What Age Should a Baby be Vaccinated?

The question thus propounded contains in strictness two separate interrogations within itself: first, whether a child should or should not be submitted to vaccination? and, secondly, if the answer to this be affirmative, at what age the inoculation should be performed?

The first question is one of those fiercely debated and disputed controversies in which neither side seems to make any great progress in its attempts to convert the other. The majority of the inhabitants of Great Britain believe that vaccination has some protective influence against smallpox: a small minority deny this vehemently, and regard vaccination as unspeakably horrible. Statistics about smallpox epidemics are freely quoted to support both of these exactly opposite contentions. Royal Commissions have sat and reported, but still there is no semblance of any common agreement about the subject. In what follows we shall for our present purpose avoid statistics entirely, and technical matters as far as possible; not because there is any lack of scientific and mathematical proof of the views expressed, but merely because the object in view is to give a comprehensible and unvarnished account of the truth as we conceive of it.

The object of vaccination is to diminish, as far as possible, the liability of the vaccinated person to contract smallpox. In modern times smallpox has become, in Britain, a very uncommon disease; but up to two or three generations ago it was exceedingly common, and its victims were either killed outright or disfigured for life. Since vaccination became compulsory, smallpox has ceased to be a cause of serious mortality amongst us. The advocates of vaccination ascribe this to the beneficent effects of that process; their opponents ascribe it to the general improvement of sanitation which has taken place during the same period. The vaccinationists point to the experience of many uncivilised communities where smallpox, introduced by European colonists, invaders, or merchant adventurers, has frequently wrought fearful havoc among native populations highly susceptible to its ravages. In the Philippine Islands, for instance, smallpox caused thousands of deaths about the beginning of this century. Compulsory vaccination was introduced by the Americans, who now govern the islands; in a year or two smallpox was controlled, and in a year or two more it was to all intents exterminated. Yet the population at large is just as indifferent to the elements of hygiene and sanitation as it was before. In Africa many illustrations of the same kind have been afforded. Several of the native tribes have been decimated by smallpox since they first came in contact with Europeans; the Masai of British East Africa are an instance in point, if one is wanted, and in Madagascar the same thing has happened under French rule. Vaccination has been followed by the almost total disappearance of the smallpox epidemics; and the significant thing is that the tribes themselves, after a short experience, have been so thoroughly convinced of the value of vaccination that they travel voluntarily long distances to be vaccinated. Yet these naked savages live still precisely the same lives as their forefathers have done for centuries, quite innocent of sanitation even of the crudest kind.

The anti-vaccinationists are firmly convinced that to inoculate "matter" of any kind is a filthy, disgusting, and entirely useless proceeding. The use of "matter" for any therapeutic purpose would certainly be repellent to delicate susceptibilities; though a large proportion of the wage-earning population do in fact daily swallow a great deal of it from decayed teeth and suppurating gums. Still, it is easy to grant that, if vaccination really did involve the use of "matter," only the great benefits which are claimed for it could reconcile fastidious people to its use. But vaccine lymph is not "matter" at all in the ordinary sense of that word. Even if it were, it would still be vastly less "disgusting" than the terrible disease which it is intended to prevent.

But such arguments as to whether calf lymph is or is not loathsome, or filthy, or any other of the adjectives so freely used about it by anti-vaccinationists are of small importance compared with the question of whether or not vaccination protects against smallpox. The experts speak with one voice on this matter; they are unanimous in holding that it does so protect vaccinated persons for a space of some years against smallpox. There are, it is true, a handful of medical men who do not admit this. But not a single one of them has any scientific reputation to risk; not a single one of them is regarded as a man of eminence in his profession; and, to put it bluntly, this handful are noted "cranks." With these few exceptions the medical profession of all degrees, from Cavendish Square to Bermondsey, and from Land's End to John o' Groats, believes firmly and honestly that vaccination is a powerful aid in the prevention of smallpox. The highest experts—namely, the medical officers of smallpox hospitals—believe this implicitly; the public health experts, into whose hands the work of checking each outbreak falls, are just as positive about it; and the general practitioners in all parts of the kingdom believe it too. Nay more, the medical experts of the entire world believe it as well, and look upon the discovery of vaccination as one of the peculiar honours of English medicine.

This expert consensus would be enough for most people. If every architect in England said the Monument was in a dangerously unsafe condition, the houses in the neighbourhood would be shunned until it had been repaired. If every engineer in

London said that Holborn Viaduct was unsafe for motor 'buses the police would promptly close it for such traffic. But because this is a question affecting human health—although anti-vaccinationists allege that the profession is influenced by the economic benefit vaccination confers upon it—there is a section of the population which considers itself fully competent to neglect expert opinion and form its own judgments, usually on a basis of blind prejudice or morbid sentimentality. This section does not hesitate to bandy and to believe the charge that the medical profession supports vaccination because some of its members make a number of half-crown fees out of it! Such a frame of mind speaks volumes for the charity of those who possess it, to say nothing more. The charge is one which is best answered by those who see the medical profession at work; by the inmates of hospital wards, or other sick people in humble circumstances; by the clergy; and by the members of the nursing profession.

Another objection often urged against the performance of vaccination is that diseases are often inoculated at the same time. It is not possible to say that this never occurs; but it is so extraordinarily rare that the contingency need not alarm the most nervous mother. Fifty years or more ago, when arm to arm vaccination was practised, such transmission of disease was, it is true, not so very, very rare as it is now. But as performed in the twentieth century the risk of vaccination is certainly no greater than—if, indeed, as great as—the risk of pricking or scratching oneself with a needle or any other sharp pointed thing.

For people of plain common sense the experience of several generations of medical practice is sufficient. That experience is in favour of vaccination, and of revaccination at intervals to renew the protection. Vaccination is not held up as an ideal or an absolutely certain preventive. In time to come bacteriologists may give us a better one. But, so far as we have as yet got, vaccination is our most valuable guarantee against smallpox, and to neglect its enforcement is to plunge the whole community, and especially its unvaccinated sections, into grave danger of a serious outbreak some day or other.

Let us come, then, to consider the second part of the question, When should an infant be vaccinated? Speaking generally, the answer is, the younger the better. To begin with, very young infants almost certainly do not feel pain or discomfort so acutely as older ones do. Sentimental people may deny this, or at least deny that it can be proved. From the nature of things, no proof can be offered; but anyone who watches carefully the conduct of a very young baby, and its emotions as expressed by its features, will soon come to admit that the probability is so strong as to amount nearly to a certainty. Vaccination is not painful in its performance, though the necessary restraint upon a baby's never-ceasing restlessness may make it cry. Nor is the after-affect (when the vaccination " takes ") as a rule very uncomfortable, though it sometimes is. But undoubtedly tiny babies pay less attention to, and are less disturbed by, a vaccinated arm or leg than do older children; and, therefore, it is quite a wise plan to have the vaccination done in the first month of life.

Not only do little babies pay less attention to a vaccination bleb than older ones, but they also " take " less extensively on the average. The value of the protection secured does not depend, we may remark, upon the intensity of the local reaction. Another advantage of early vaccination is that the " monthly nurse " is still in the house, if one has been employed, and can supervise the necessary arrangements, hold the baby, and generally assist the doctor much better than the mother.

The drawback to early vaccination is that every subsequent ailment or illness from the time of vaccination onwards is ascribed to the inoculation. The rashes of congenital syphilis, eczema, intertrigo, etc., not uncommonly develop between the third and sixth weeks of life; should they do so, they are certain to be attributed to vaccination. There can be no doubt that in the vast majority of cases where syphilis is asserted to have been inoculated with vaccination, the disease has in reality been congenital—derived, that is, from one or other parent, but not developed in any form to attract attention until some weeks or months after birth. In the same way sweat rashes, rashes due to wetted pilches, digestive disturbances due to imperfect or improper diet, and all kinds of ill-health whatever and wherever and whenever occurring, are liable to be confidently ascribed to vaccination by those who have any prejudice in the matter.

If, therefore, there is the slightest suspicion of syphilis or any other disorder, or if the baby is premature or delicate, or if it is not thriving as it should do, the wiser plan is to postpone vaccination from the first to the second month. Only very exceptional circumstances should lead to any longer delay than this; and no circumstances at all (except an actual attack of smallpox) should stand in the way of every child receiving the protection and benefit which vaccination confers.

